# Associations between the standardized uptake value of ^18^F-FDG PET/CT and the prognostic factors of invasive lobular carcinoma: in comparison with invasive ductal carcinoma

**DOI:** 10.1186/s12957-015-0522-9

**Published:** 2015-03-15

**Authors:** Na Young Jung, Sung Hun Kim, Bo Bae Choi, Sung Hoon Kim, Mi Sook Sung

**Affiliations:** Department of Radiology, Bucheon St. Mary’s Hospital, College of Medicine, The Catholic University of Korea, 327 Sosa-ro, Wonmi-gu, Bucheon-si, Gyeonggi-do 420-717 Republic of Korea; Department of Radiology, Seoul St. Mary’s Hospital, College of Medicine, The Catholic University of Korea, 222 Banpo-daero, Seocho-gu, Seoul 137-701 Republic of Korea; Department of Radiology, Chungnam National University Hospital, 282 Muhwha-ro Jung-gu, Daejeon, 301-721 Republic of Korea

**Keywords:** Breast neoplasm, Invasive lobular carcinoma, Invasive ductal carcinoma, Positron emission tomography/computed tomography, Estrogen receptor, Progesterone receptor

## Abstract

**Background:**

The aims of this study were to evaluate the associations between the maximum standardized uptake value (SUVmax) and prognostic factors in invasive lobular carcinoma (ILC) and to compare these results with those in invasive ductal carcinoma (IDC).

**Methods:**

The study included pathologically confirmed ILCs (*n* = 32) and IDCs (*n* = 73). We retrospectively evaluated the preoperative ^18^F-fluorodeoxyglucose positron emission tomography/computed tomography (^18^F-FDG PET/CT) and measured the SUVmax. The pathologic results were reviewed regarding the size, histological type, histological grade, estrogen receptor (ER) and progesterone receptor (PR) status, human epidermal growth factor receptor 2 (HER2), epidermal growth factor receptor (EGFR), and Ki-67 of the primary tumor. We also compared the associations between the SUVmax and prognostic factors.

**Results:**

The mean SUVmax of the ILCs was significantly lower compared with that of the IDCs (*P* = 0.032). The SUVmax increased with tumor grade (*P* < 0.001) and was higher with ER negativity compared with ER positivity (*P* = 0.007) in IDC. The SUVmax was higher with EGFR positivity compared with EGFR negativity (*P* = 0.013) in IDC and higher with Ki-67 positivity compared with Ki-67 negativity in IDC and ILC (*P* < 0.001 and *P* = 0.002, respectively). The SUVmax was not significantly different regarding PR or HER2 for both tumor groups. The correlation between the tumor size and the SUVmax was demonstrated for IDCs (*r* = 0.57), but not for ILCs (*r* = 0.25).

**Conclusions:**

The SUVmax was significantly different according to the tumor grade, ER, EGFR, and Ki-67 for IDCs. The SUVmax exhibited a positive association with Ki-67 in ILC; however, it was not significantly different with other factors, which suggests that the role of ^18^F-FDG PET/CT may be limited in ILC.

## Background

Breast cancer is a heterogeneous disease with a broad range of therapeutic responses, recurrence risk, and overall prognosis [1]. Predicting the prognosis of breast cancer is very important to determine the direction of treatment. The conventional prognostic factors include the tumor nuclear grade, tumor size, and presence of lymph node metastasis. The immunohistochemical prognostic factors include hormone receptors, such as estrogen receptor (ER), progesterone receptor (PR) and human epidermal growth factor receptor 2 (HER2), and the Ki-67 proliferation index [2].

^18^F-fluorodeoxyglucose positron emission tomography/computed tomography (^18^F-FDG PET/CT) has become an increasingly important tool in the evaluation of patients with invasive breast cancer by revealing the functional properties of breast tumors [1]. ^18^F-FDG uptake in tumors is affected by various mechanisms, such as glucose transporter-1 (GLUT1) expression, hexokinase activity in tumor cells, tumor vascularity, tumor necrosis, the density of tumor cells, and the mitotic activity index [3]. The maximum standardized uptake value (SUVmax) measured with FDG PET is a sensitive indicator for metabolic activity in breast cancer [4,5], which can be used to assess tumor aggressiveness and is associated with prognostic factors, such as the histological type, histological grade, immunohistochemical factors, and proliferation index [2-4,6-10]. These studies have primarily included invasive ductal carcinoma (IDC), which comprises 72% to 80% of all invasive breast cancers [11]. Invasive lobular carcinoma (ILC) is the second most common breast cancer after IDC and accounts for 5% to 15% of all breast cancers [11,12], but there is a lack of data associating the SUVmax and prognostic factors in ILC in the existing literature. ILCs have exhibited low FDG uptake in previous studies [3,4,7-9,13-15].

To our knowledge, no study has associated FDG uptake with prognostic factors in this specific type of breast cancer. Thus, the aims of this study were to associate the SUVmax with histopathological and immunohistochemical prognostic factors in ILC and to compare these findings with those in IDC.

## Methods

### Patients

Institutional review board approval was obtained for this retrospective study. The informed consent was waived. We reviewed medical records and identified 32 patients with ILC between January 2004 and June 2012 and 73 patients with IDC in 2009. All patients underwent preoperative ^18^F-FDG PET/CT, in addition to breast magnetic resonance imaging and subsequent surgical diagnosis and staging.

### ^18^F-FDG PET/CT imaging

All patients underwent preoperative ^18^F-FDG PET/CT examinations using a dedicated PET/CT scanner with two-slice CT (Siemens Biograph Classic; Siemens Medical Solutions, Knoxville, TN, USA) (*n* = 60) or with a PET/CT scanner with 40-slice CT (Siemens Biograph TruePoint; Siemens Medical Solutions, Knoxville, TN, USA) (*n* = 45). The patients were asked to fast for a minimum of 6 h prior to the examination. Serum glucose levels were measured to ensure euglycemia (blood glucose level <130 mg/dl). Approximately 370 to 550 MBq of ^18^F-FDG were then injected with saline infusion. Following 60 min of bed rest after the injection, the PET scans were obtained. Seven to eight bed positions were acquired, with an acquisition time of 2 min each. All patients were in a supine position with their arms raised during the PET/CT scanning. Noncontrast CT scanning was initiated at the orbitomeatal line and progressed to the upper thigh (30 mAs; 130 kV; slice thickness 5 mm); the corresponding PET imaging immediately followed over the same body region. The CT data were used for attenuation correction, and the images were reconstructed using the standard ordered subset expectation maximization (OSEM; two iterations, eight subsets) algorithm.

### Imaging interpretation

The ^18^F-FDG PET/CT imaging data were interpreted retrospectively by a nuclear medicine physician. All PET/CT images were reviewed at a workstation with fusion software (Syngo; Siemens Medical Solutions, Knoxville, TN, USA), which provided multiplanar reformatted images and displayed PET images, CT images, and PET/CT fusion images. A nuclear medicine physician reviewed the PET/CT images, and the interpretation was visually performed. For semi-quantitative analysis, SUVmax of ^18^F-FDG was measured by visually placing the regions of interest (ROIs) around the primary cancer mass that had perceptible ^18^F-FDG uptake. For compensation of two different PET/CT scanner types, the mean liver SUV values were obtained for all patients. The tumor/liver SUV ratio was calculated and compared between two groups using the different PET/CT scanner types. The tumor/liver SUV ratio was not statistically different according to the PET/CT scanner types (2.11 ± 1.89 for biograph classic with two-slice CT *versus* 1.75 ± 1.26 for biography true point with 40-slice CT, *P* = 0.299).

### Histopathological analysis

We obtained the histopathological findings, including the size of invasive cancer, histological type, histological grade, ER and PR status, HER2, epidermal growth factor receptor (EGFR), and Ki-67 of the primary tumor by reviewing the pathology reports. Histopathological grading was performed using the Elson-Elis method, in which tubule formation, pleomorphism, and mitotic counts are scored 1 to 3 points. The cases scored within the 3 to 5 range were designated as grade 1, within 6 to 7 as grade 2, and within 8 to 9 as grade 3. Immunohistochemistry was used to assess the expression of the following molecular markers: ER, PR, HER2, EGFR, and Ki-67. ER and PR positivity was defined as the presence of 10% or more positively stained nuclei in ten high-power fields. The intensity of HER2 immunohistochemical (IHC) staining was scored as 0, 1+, 2+, or 3+. The tumors with 3+ were classified as positive, whereas 0 and 1+ were negative. For an HER2 score of 2+, fluorescence *in situ* hybridization (FISH) was used to determine HER2 positivity. EGFR was considered positive if membrane staining was observed. A Ki-67 of ≥15% was considered positive expression, which is comparable to 2011 Galen Consensus [16] and previous other studies of our institution [2,17].

### Statistical analysis

Continuous variables are shown as the mean ± standard deviation (SD) with or without median (interquartile range), and the categorical variables are presented as the frequency and percentage. The differences between patient age and histopathological findings in ILCs and IDCs were compared using the Wilcoxon rank sum and Kruskal-Wallis tests. The association between the histopathological variables (for example, histological type, histological grade, ER and PR status, HER2, EGFR, and Ki-67 of the primary tumor) and the SUVmax were compared in each group of total carcinomas, ILCs, and IDCs using the Wilcoxon rank sum and Kruskal-Wallis tests. The correlations between the tumor size and the SUVmax were determined by the Spearman correlation coefficient and *P* value.

Significance was established at *P* < 0.05. The evaluation of the results was performed using the SAS system for Windows V 9.1 (SAS Institute, Cary, NC, USA).

## Results

### Patients and tumor characteristics

The clinical and histopathological data are summarized in Table 1. The tumor size was significantly larger in the ILC group compared with the IDC group (2.64 ± 1.13 *vs.* 2.08 ± 1.69 cm; *P* = 0.002). Compared with IDCs, ILCs were more likely to be positive for ER (93.6% *vs*. 75.3%; *P* = 0.031) and PR (83.9% *vs*. 61.1%; *P* = 0.023) and less likely to overexpress HER2 (6.7% *vs*. 24.6%; *P* = 0.039) and Ki-67 (18.5% *vs*. 51.4%; *P* = 0.003).

**Table 1:** **Clinical and histopathological characteristics of ILC and IDC patients**

	**Total (** ***n*** **= 105)**	**ILC (** ***n*** **= 32)**	**IDC (** ***n*** **= 73)**	***P*** **value**
Age (*n* = 105; 32, 73)^a^	52 ± 9.46	53.28 ± 9.52	51.44 ± 9.44	0.529
Tumor size (*n* = 104; 31, 73)^a^	2.25 ± 1.56	2.64 ± 1.13	2.08 ± 1.69	0.002
Tumor Grade (*n* = 105; 32, 73)^a^				0.709
Grade 1	35 (33.3)	9 (28.1)	26 (35.6)	
Grade 2	53 (50.5)	18 (56.3)	35 (48.0)	
Grade 3	17 (16.2)	5 (15.6)	12 (16.4)	
ER (*n* = 104; 31, 73)^a^				0.031
Negative	20 (19.2)	2 (6.5)	18 (24.7)	
Positive	84 (80.8)	29 (93.6)	55 (75.3)	
PR (*n* = 103; 31, 72)^a^				0.023
Negative	33 (32.0)	5 (16.1)	28 (38.9)	
Positive	70 (68.0)	26 (83.9)	44 (61.1)	
HER2 (*n* = 91; 30, 61)^a^				0.039
Negative	74 (81.3)	28 (93.3)	46 (75.4)	
Positive	17 (18.7)	2 (6.7)	15 (24.6)	
EGRF (*n* = 98; 27, 71)^a^				0.221
Negative	82 (83.7)	25 (92.6)	57 (80.3)	
Positive	16 (16.3)	2 (7.4)	14 (19.7)	
Ki-67 (*n* = 99; 27, 72)^a^				0.003
Negative	57 (57.6)	22 (81.5)	35 (48.6)	
Positive	42 (42.4)	5 (18.5)	37 (51.4)	

Note: The age and tumor size were shown in mean ± standard deviation (SD) and other values were presented as number of cases and percentage in parenthesis.

^a^(*n* = number of total cases; number of ILC, number of IDC).

EGRF, epidermal growth factor receptor; ER, estrogen receptor; HER2, human epidermal growth factor receptor 2; IDC, invasive ductal carcinoma; ILC, invasive lobular carcinoma; PR, progesterone receptor.

The patient’s age, tumor grade, and EGFR of the primary tumor were not different between the ILCs and IDCs.

### SUVmax and histopathological variables

**Figure 1:**
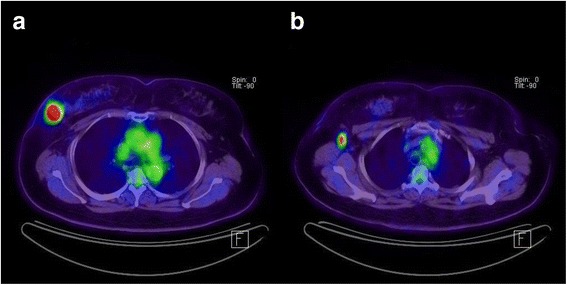
**A 57-year-old woman with invasive ductal carcinoma in the right breast. (a)** ^18^F-FDG PET/CT shows focal FDG uptake in the right upper outer breast with a maximum standardized uptake value (SUVmax) measured at 13.6. The tumor grade was III. On immunohistochemical study, ER and PR were negative and Ki-67 and EGFR were positive. **(b)** ^18^F-FDG PET/CT at axillary level shows focal FDG uptake in the right axillary lymph node with a SUVmax measured at 5.8. On histologic examination, right axillary lymph node metastases were noted in 7 of 16 dissected lymph nodes. EGFR, epidermal growth factor receptor; ER, estrogen receptor; ^18^F-FDG PET/CT, ^18^F-fluorodeoxyglucose positron emission tomography/computed tomography; PR, progesterone receptor.

**Figure 2:**
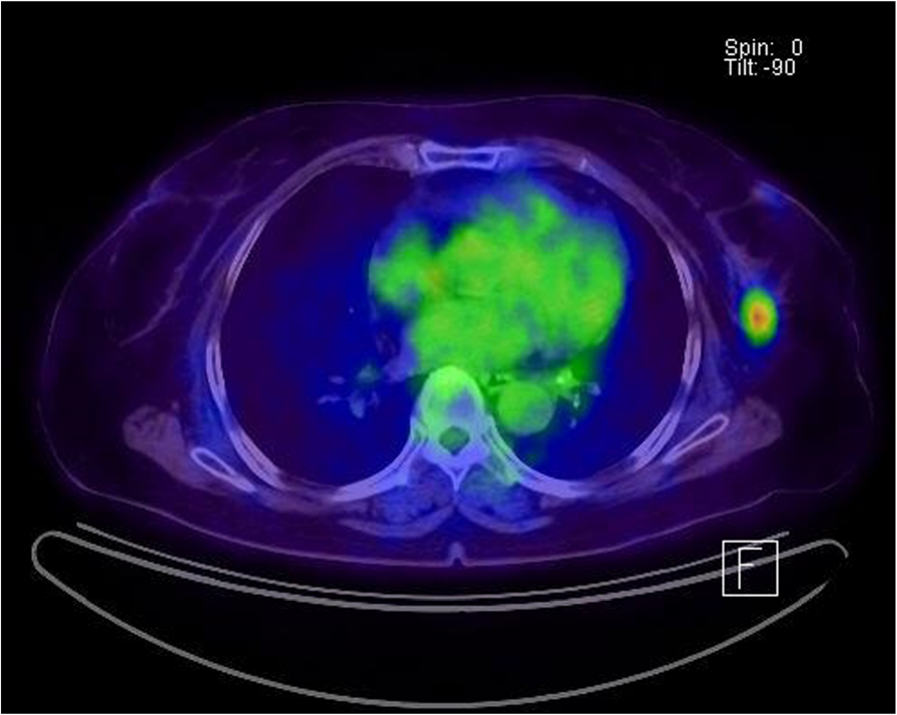
**A 76-year-old woman with invasive lobular carcinoma in the left breast.** ^18^F-FDG PET/CT shows focal FDG uptake in the left mid outer breast with a maximum standardized uptake value (SUVmax) measured at 5.3. On immunohistochemical study, Ki-67 was positive. ^18^F-FDG PET/CT, ^18^F-fluorodeoxyglucose positron emission tomography/computed tomography.

The association between the SUVmax and the histopathological variables are summarized in Table 2. The mean SUVmax of the ILCs (1.99 ± 1.72) was significantly lower compared with the IDCs (3.91 ± 3.99) (*P* = 0.032). The SUVmax increased with tumor grade (*P* < 0.001) and was higher with ER negativity compared with ER positivity (6.39 ± 5.06 *vs*. 3.09 ± 3.23; *P* = 0.007) in the IDC group (Figure 1). The SUVmax was higher with EGFR positivity compared with EGFR negativity (6.92 ± 5.55 *vs*. 3.26 ± 3.22; *P* = 0.013) in the IDC group (Figure 1) and higher with Ki-67 positivity compared with Ki-67 negativity (5.58 ± 4.66 *vs*. 2.19 ± 2.15, IDC, *P* < 0.001; 4.18 ± 0.88 *vs*. 1.45 ± 1.22, ILC, *P* = 0.002) in both the IDC and ILC groups (Figures 1 and 2). The SUVmax was not significantly different according to PR or HER2 for both tumor groups.

**Table 2:** **Association between clinical pathological variables and SUVmax values**

	**SUVmax**		
	**Total (** ***n*** **= 105)**	**ILC (** ***n*** **= 32)**	**IDC (** ***n*** **= 73)**
	**Mean ± SD**	**Mean ± SD**	**Mean ± SD**
	**Median (interquartile range)**	**Median (interquartile range)**	**Median (interquartile range)**
Cancer type (*n* = 105; 32, 73)^a^			
ILC (*n* = 32)	1.99 ± 1.72		
	1.70 (0.50 to 3.05)		
IDC (*n* = 73)	3.91 ± 3.99		
	2.30 (1.40 to 6.10)		
*P* value	0.032		
Tumor Grade (*n* = 105; 32, 73)^a^			
Grade 1 (*n* = 35; 9, 26)^a^	2.07 ± 2.02	1.63 ± 1.36	2.22 ± 2.21
	1.70 (1.10 to 2.40)	1.90 (0.00 to 2.40)	1.60 (1.20 to 2.30)
Grade 2 (*n* = 53; 18, 35)^a^	2.97 ± 3.37	1.76 ± 1.54	3.59 ± 3.88
	2.20 (1.00 to 3.60)	1.60 (0.00 to 3.20)	2.30 (1.20 to 5.30)
Grade 3 (*n* = 17; 5, 12)^a^	7.02 ± 4.31	3.46 ± 2.46	8.51 ± 4.07
	6.70 (4.50 to 8.40)	2.80 (1.50 to 5.30)	7.25 (6.05 to 11.90)
*P* value	<0.001	0.393	< 0.001
ER (*n* = 104; 31, 73)^a^			
Negative (*n* = 20; 2, 18)^a^	5.84 ± 5.10	0.80 ± 1.13	6.39 ± 5.06
	5.55 (1.90 to 7.85)	0.80 (0.00 to 1.60)	5.95 (2.30 to 8.40)
Positive (*n* = 84; 29, 55)^a^	2.77 ± 2.83	2.14 ± 1.72	3.09 ± 3.23
	1.95 (1.20 to 3.45)	1.80 (1.00 to 3.20)	2.10 (1.30 to 4.70)
*P* value	0.010	0.257	0.007
PR (*n* = 103; 31, 72)^a^			
Negative (*n* = 33; 5, 28)^a^	4.82 ± 5.00	0.92 ± 0.85	5.52 ± 5.11
	2.30 (1.30 to 7.30)	1.30 (0.00 to 1.60)	4.90 (1.55 to 7.55)
Positive (*n* = 70; 26, 44)^a^	2.71 ± 2.41	2.27 ± 1.76	2.97 ± 2.71
	2.10 (1.30 to 3.50)	2.00 (1.00 to 3.40)	2.15 (1.40 to 4.10)
*P* value	0.157	0.089	0.064
HER2 (*n* = 91; 30, 61)^a^			
Negative (*n* = 74; 28, 46)^a^	3.13 ± 3.51	2.03 ± 1.64	3.80 ± 4.14
	2.10 (1.20 to 3.70)	1.75 (1.00 to 3.05)	2.15 (1.30 to 5.80)
Positive (*n* = 17; 2, 15)^a^	4.59 ± 4.16	2.65 ± 3.75	4.85 ± 4.26
	3.50 (1.80 to 6.90)	2.65 (0.00 to 5.30)	3.50 (1.80 to 7.30)
*P* value	0.113	0.900	0.230
EGRF (*n* = 98; 27, 71)^a^			
Negative (*n* = 82; 25, 57)^a^	2.85 ± 2.86	1.90 ± 1.44	3.26 ± 3.22
	2.05 (1.20 to 3.50)	1.80 (1.00 to 2.90)	2.10 (1.30 to 4.80)
Positive (*n* =16; 2, 14)^a^	6.39 ± 5.45	2.65 ± 3.75	6.92 ± 5.55
	5.55 (2.40 to 7.25)	2.65 (0.00 to 5.30)	5.95 (2.60 to 7.30)
*P* value	0.006	0.815	0.013
Ki-67 (*n* = 99; 27, 72)^a^			
Negative (*n* = 57; 22, 35)^a^	1.90 ± 1.87	1.45 ± 1.22	2.19 ± 2.15
	1.60 (1.00 to 2.30)	1.65 (0.00 to 2.30)	1.60 (1.10 to 2.30)
Positive (*n* = 42; 5, 37)^a^	5.41 ± 4.40	4.18 ± 0.88	5.58 ± 4.66
	4.75 (2.30 to 7.30)	4.30 (3.40 to 4.70)	5.30 (2.20 to 7.30)
*P* value	< 0.001	0.002	< 0.001

^a^(*n* = number of total cases; number of ILC, number of IDC).

EGRF, epidermal growth factor receptor; ER, estrogen receptor; HER2, human epidermal growth factor receptor 2; IDC, invasive ductal carcinoma; ILC, invasive lobular carcinoma; PR, progesterone receptor.

A correlation between the tumor size and the SUVmax was identified in IDCs (*r* = 0.57), but not ILCs (*r* = 0.25) (Table 3).

**Table 3:** **Correlation between Tumor size and SUVmax**

	**Total**	**ILC**	**IDC**
	**(** ***n*** **= 105)**	**(** ***n*** **= 32)**	**(** ***n*** **= 73)**
Spearman correlation coefficient	0.40	0.25	0.57
*P* value	<0.001	0.1787	<0.001

IDC, invasive ductal carcinoma; ILC, invasive lobular carcinoma; SUVmax, maximum standardized uptake value.

## Discussion

The objectives of this study were to associate the SUVmax with prognostic factors in ILC and to compare these results with the associations in IDC.

In the present study, the tumor size was significantly larger in the ILC group compared with the IDC group. Compared with IDCs, ILCs were more likely to be positive for ER and PR and less likely to overexpress HER2 and Ki-67. The results of the present study are consistent with previous studies [12,18,19].

In the present study, because the distributions of SUVmax values were skewed, the association between clinical/histopathological variables and SUVmax values was performed by the Wilcoxon rank sum test. Our study revealed that the mean SUVmax of ILCs was significantly lower compared with that of IDCs, which was consistent with previous results [3,4,7-9,13-15]. The lower FDG uptake of ILC was explained by diffuse infiltrative growth patterns, a low tumor cell density, a low level of GLUT1 expression, and a decreased proliferation rate [3,13,15]. The SUVmax was significantly different according to the tumor grade, ER, EGFR, and Ki-67 for IDCs; however, only Ki-67 was significantly different for ILCs in the present study. The SUVmax was not significantly different according to PR or HER2 for both tumor groups. These results for IDCs were consistent with previous studies regarding breast cancers in which the SUVmax was significantly higher in patients with tumors with negative ER and grade III [2,4,6-10,14,20]. There have been some controversies regarding the association between the SUVmax and the negativity of PR [2,4,6,8-10,14,20] and the positivity of EGFR [2,9]. There was no association between the SUVmax and HER2 status in IDC or ILC in the present study, and these findings are nearly identical to previous results [2,4,6,8-10,14,20]; however, the current findings are in disagreement with previous results that indicated a positive association between the SUV max and HER2 positivity [10,14].

In contrast with IDC, the SUVmax showed a positive association only with Ki-67 in ILC in the present study. The association between the SUVmax and the Ki-67 proliferation index in breast carcinoma has been reported in previous studies [2,4,6,7]; however, the majority (84% to 100%) of cases in these studies had IDCs. To the best of our knowledge, no previous report has evaluated the association between the SUVmax and prognostic factors in ILC. The role of ^18^F-FDG PET/CT as an imaging biomarker to predict the prognosis in breast cancers has been demonstrated in many papers [2-4,6-10,14]. We have also demonstrated the role of ^18^F-FDG PET/CT as an imaging biomarker for the prognostic prediction of breast cancers in IDC. However, the role of ^18^F-FDG PET/CT may be assumed to be limited in ILC.

The correlation between the tumor size and the SUVmax was demonstrated in IDCs, but not in ILCs in the present study. The IDC results in the present study were similar to previous studies that reported a positive relationship between SUVmax and tumor size in breast cancer [2,6,7,9,10,14], but not consistent with previous result that indicated there was no correlation between the SUVmax and T stage [8].

The limitations of the current study included the small number of cases, especially for ILC, and the retrospective design. Furthermore, the reviewers were aware that all cases had breast cancer, although they did not know whether the diagnosis was IDC or ILC, which may have resulted in selection bias. Because the review of histopathological report was also retrospective, the data file included a certain amount of missing values. The high rate of missing values for HER2 was due to lack of FISH results for cases showing HER2 score of 2+ on IHC staining, because the FISH was not a routine exam but an ancillary test based on request by clinicians. The missing values had less than 15%, so the list-wise deletion was used for missing data.

## Conclusions

In conclusion, the mean SUVmax of ILCs was significantly lower compared with that of IDCs. The SUVmax was significantly different according to the tumor grade, ER, EGFR, and Ki-67 in IDCs, but only for Ki-67 in ILCs. Therefore, the role of preoperative ^18^F-FDG PET/CT in terms of prognosis prediction may be more difficult in ILCs than IDCs.

## Abbreviations

EGFR: Epidermal growth factor receptor
ER: Estrogen receptor
^18^F-FDG PET/CT: ^18^F-fluorodeoxyglucose positron emission tomography/computed tomography
GLUT1: Glucose transporter-1
HER2: Human epidermal growth factor receptor 2
IDC: Invasive ductal carcinoma
ILC: Invasive lobular carcinoma
PR: Progesterone receptor
SUVmax: Maximum standardized uptake value
